# Supplementary material for: MetaRibo-Seq measures translation in microbiomes
Source: Nat Commun. 2020 Jun 29;11:3268. doi: 10.1038/s41467-020-17081-z (PMC7324362; doi:10.1038/s41467-020-17081-z)
Supplement: Supplementary file 10 — Supplementary Data 7 [file 41467_2020_17081_MOESM10_ESM.zip › File2/Confidence_High_Taxonomy/84844_out.krona.html]

Javascript must be enabled to view this page.

members
magnitude
magnitudeUnassigned
count
unassigned
taxon
rank

84844\_out

9

8

SRS013687\_contig\_number\_contig-100\_42047.86990SRS017521\_contig\_number\_contig-100\_9257.207898SRS045713\_contig\_number\_contig-100\_9663.73072SRS1041036\_contig\_number\_contig-100\_4136.4137SRS1041136\_contig\_number\_17435SRS143417\_contig\_number\_21509SRS144537\_contig\_number\_34027SRS148721\_contig\_number\_29034

1
2
superkingdom

phylum
1
1239

class
1
186801

order
1
186802

1
216572
family

1
459786
genus

species

SRS104084\_contig\_number\_contig-100\_10.87293
1
876091
